# Measurement invariance on two self-report instruments for men and women with borderline personality disorder

**DOI:** 10.1186/s40479-025-00296-1

**Published:** 2025-05-12

**Authors:** Leonie Strunk, Kathrin Dreyße, Christoph Kröger

**Affiliations:** 1 Institute of Psychology, University of Hildesheim, Universitätsplatz 1, Hildesheim, 31141 Germany; 2 Schön Clinic Bad Bramstedt, Birkenweg 10, Bad Bramstedt, 24576 Germany

**Keywords:** Borderline personality disorder, Measurement invariance, IES-27, Emotion dysregulation, BSL-23, Gender bias

## Abstract

**Background:**

In clinical practice and research, self-report instruments are frequently used for assessing the severity of borderline personality disorder (BPD) symptomatology experienced by men and women. Men with BPD are often underrepresented in samples used to evaluate self-report questionnaires. Measurement invariance (MI) is used to examine whether self-report questionnaires determine the same latent construct across groups or varying conditions (e.g., measurement occasions).

**Methods:**

The present study investigated measurement invariance for two self-report measures of BPD features: the *Borderline Symptom List* (BSL-23) and the *Impulsivity and Emotion Dysregulation Scale* (IES-27). An inpatient sample of *N* = 3507 individuals (*n* = 560 males) was used to test for measurement variance between males and females, and over time from pre- to post-treatment.

**Results:**

Confirmatory factor analysis results supported a unidimensional structure for the BSL-23 and a three-factor model for the IES-27. Both instruments were found to be measurement invariant with regard to sex and time.

**Conclusions:**

The results suggest that the BSL-23 and IES-27 can be used to assess BPD symptoms in men and women, as well as to assess treatment effects at admission and at the end of treatment.

**Supplementary Information:**

The online version contains supplementary material available at 10.1186/s40479-025-00296-1.

## Introduction

The community point prevalence of borderline personality disorder (BPD) in adults is estimated at between 0.7% and 2.7%, while patients with BPD constitute around 12% of the outpatient and 22% of the inpatient psychiatric population [1]. Prevalence studies indicated nearly the same rate of BPD among men and women in community samples [2–4], whereas the male-to-female ratio in clinical settings stands at 1:3 [5]. Data from a Swedish health and administrative register showed that only 15% of the patients diagnosed with BPD were men [6]. Gender difference in prevalence rates may reflect true group difference, as men with BPD may be less inclined to seek treatment or are more commonly found in other treatment settings, such as criminal justice systems [7]. Alternatively, this may reflect some form of “gender bias” in the diagnostic procedures of BPD [6]. Gender bias in diagnosis could occur based on different factors, such as biased diagnostic criteria, biased diagnostic thresholds, biased application of diagnostic criteria, or biased measurement instruments [8]. Studies with case vignettes, for example, portend that clinicians judge BPD-related problems differently in men than in women [9, 10]. For measurement instruments, one way of investigating whether the expression or understanding of BPD-related problems differs between men and women is to analyze measurement invariance (MI). MI is given when items (manifest variables) assess the same latent construct across different populations or conditions (e.g., measurement points; [11]).

Structured interviews are the gold standard for diagnosing BPD in clinical practice and research. A lack of MI was found in two of nine BPD criteria in an epidemiological sample assessed by structured interviews [12]. Affective instability was less frequently reported by men at the same BPD-factor level. For impulsivity, factor loadings and thresholds were moderated by sex and by the interaction of age and sex; men reported more impulsivity than women, and this difference increased disproportionately with age [12]. By investigating the differential item functioning (DIF) of the BPD criteria in structured interviews with an inpatient sample, it was observed that men were more likely than women to endorse the criteria impulsivity and uncontrolled anger at the same parameter value on the latent BPD factor [13]. In a mixed sample of outpatients and students, the structured interview was invariant with respect to sex [14].

Besides structured interviews, self-report questionnaires for BPD are widely used instruments in research and clinical practice for screening patients, assessing the severity of BPD symptomatology, monitoring changes during treatment, and informing clinical decision-making. In the clinical samples used to evaluate or validate self-report questionnaires, men are often under-represented, similar to their proportion in clinical settings [15, 16]. One study showed MI between men and women for a self-report measure of BPD symptomatology — namely, the *Personality Assessment Inventory Borderline Features Scale* — in a representative population sample [17]). To the best of our knowledge, no studies on self-report measures for BPD symptomatology investigate whether such measures assess the same latent factor in men and women with BPD in clinical samples.

To assess treatment effectiveness, it is crucial to calculate MI between groups as well as over time, ensuring that any observed effects may be attributed to the treatment rather than to inconsistency in the questionnaire construct. Measurement variance over time can arise due to different reasons: changes in the respondent’s internal standards of measurement (scale recalibration), changes in the respondent’s values (reprioritization), or redefinition of the target construct (reconceptualization; [18]). Longitudinal MI (before and after treatment) has only been investigated in a mixed-sex sample for the interview version of the *Zanarini Rating Scale for BPD* (ZAN-BPD; [19]). Findings suggest that the ZAN-BPD ratings were invariant across time. Only *n* = 68 males (25%) were included in the sample (*N* = 276), and separate analyses for both genders were not performed [19].

Therefore, the first aim of the present study was to investigate MI on two self-report measures that are disseminated in clinical practice. The second aim was to investigate MI for men and women over time (at admission and at the end of treatment).

## Methods

### Participants

This study was approved by the local ethics board (336). The data processing conducted was completely anonymized. We analyzed routine data collected between 2014 and 2021 from patients who were treated in a German psychosomatic clinic on the wards for personality disorders. The exclusion criteria for treatment were dementia, psychosis, current substance abuse, and suicide attempts within the last three months before admission, the reason being that all these patients need more intensive care, such as inpatient psychiatric treatment along with accompanying confinement in a closed department. At admission, all participants underwent a computer-based psychological testing procedure consisting of general psychiatric and symptom-specific questionnaires. Diagnoses were given by certified psychotherapists and psychiatrists, as well as by psychologists in post-graduate training. The German versions of the SCID-II [20] along with screening, anamnesis, and clinical behavior observation were used to diagnose personality disorders. All patients were required to fulfill the diagnosis of BPD.

The sample consisted of *N* = 3507 patients, of whom *n* = 2947 (84%) were female and *n* = 560 (16%) were male. This categorization was based on the patient’s self-reported sex at admission, which was compared with the identity card of their health insurer. The length of stay in the clinic was 58 days on average (*SD* = 21.5). Of the women, *n* = 619 (21.0%) did not complete the treatment regularly, while this was true for *n* = 99 (17.7%) of the men. Table 1 presents an overview of the participants’ socio-demographic and clinical characteristics.

### Measures

#### Impulsivity and emotion dysregulation scale (IES-27)

The IES-27 [21] measures the manifestation of clinically important impulsivity, suicidal behavior and self-injury, emotion dysregulation, and interpersonal turmoil [21]. The 27 items ask for the frequency of these behaviors in the last month on a 5-point Likert scale (0 = “not at all,” 1 = “1–2 times,” 2 = “3–10 times,” 3 = “every day,” 4 = “several times per day”). Internal consistency values ranged from ω = 0.79 to ω = 0.93 in a sample of BPD patients. A confirmatory factor analysis (CFA) suggested a bi-factor model with three specific factors (*Emotional Dysregulation*, *Relationship Distress*, and *Suicidal/ Self-injury Behavior;* [22]).

**Table 1:** Socio-demographic characteristics and additional mental disorders

Characteristics	Men (%)	Women (%)
*School education*		
In school	3 (0.6)	68 (2.4)
No school-leaving qualification	13 (2.5)	68 (2.4)
Special needs qualification	8 (1.5)	26 (0.9)
Lower-track school leaving qualification	131 (25.3)	545 (19.5)
Medium-track school leaving qualification	215 (41.5)	1233 (44.2)
University-entrance-level school-leaving qualification	137 (26.4)	791 (28.4)
No information	11 (2.1)	57 (2.0)
*Employment status*		
Unemployed	192 (37.1)	1080 (38.7)
Full time	136 (26.3)	434 (15.6)
Part time	20 (3.9)	247 (8.9)
Marginally employed	8 (1.5)	81 (2.9)
Housewife/husband/Student/In training	48 (9.3)	397 (14.2)
Pensioner	101 (19.5)	473 (17.0)
No information	12 (2.3)	76 (2.7)
*Prior outpatient treatments*		
Outpatient psychiatric	140 (51.3)	56.4)
Outpatient psychotherapeutic	362 (69.9)	1977 (70.9)
*Mental disorders*		
Affective disorders	506 (90.4)	2663 (90.4)
Social anxiety disorder	100 (17.9)	517 (17.5)
Other anxiety disorders	30 (5.4)	208 (7.1)
Somatoform disorders	48 (8.6)	201 (6.8)
Eating disorders	44 (7.9)	910 (30.9)
Posttraumatic Stress Disorder	50 (8.9)	503 (17.1)
Avoidant PD	27 (4.8)	172 (5.8)
Obsessive-compulsive PD	18 (3.2)	40 (1.4)
Histrionic PD	12 (2.1)	27 (0.9)
Other PD according to ICD-10 (F60.8)	49 (8.8)	32 (1.1)

*PTSD* Posttraumatic Stress Disorder, *PD* Personality Disorder; the table lists only those additional PDs for which the percentage was over 1% of the total sample

#### Short version of the borderline symptom list (BSL-23)

The BSL-23 [23] is a self-rated scale that assesses BPD symptomatology on 23 items. All items are rated on a 5-point Likert scale (0 = “not at all,” 1 = “a little,” 2 = “rather,” 3 = “much,” 4 = “very strong”). The period selected for the evaluation of symptoms is the previous week. Internal consistency values of the German version ranged from α = 0.94 to α = 0.97 in different samples. The original version assumed a one-factor structure based on a principal component analysis [23]. CFA of the translated version of the BSL-23 in Spanish and Mandarin confirmed the one-factor structure, although the French-language version did not [24–26].

#### Statistical analyses

The sample was randomly split into two halves, stratified by sex (men = 1; women = 0). This allowed us to conduct an exploratory factor analysis (EFA) so as to test the factor structure of the IES-27 on the first half. We used diagonally weighted least squares (WLSMV) to estimate model parameters throughout our analyses because of the ordinal data structure [27]. EFA with oblique (geomin) rotation was conducted sequentially across models with one to four factors. Factor retention was guided by parallel analysis, examination of the scree plot, root mean-square of approximation (RSMEA), and clinical meaningfulness of the extracted factors. During the EFA, items with negative or no loadings or with significant cross-loadings on multiple factors were dropped. Using WLSMV as an estimator, a CFA was conducted on the second half of the sample to subsequently assess reproducibility of the EFA-derived solution. The criteria for adequate model fit in CFA included RMSEA, comparative fit index (CFI), and Tucker-Lewis index (TLI). RMSEA values ≤ 0.050 indicate a close model fit, while values between 0.050 and 0.080 indicate an adequate model fit. Values greater than 0.10 indicate poor fit. For CFI and TLI, an adequate model fit is achieved when CFI and TLI are ≥ 0.900, whereas values ≥ 0.950 indicate a good model fit [28]. For BSL-23, we assumed a one-factor model based on consistent results in previous studies, and reviewed this in a CFA [25, 26]. We assessed MI of the final factor solution between men and women and for both sexes over time by comparing increasingly constrained models in a CFA framework. First, the configural model assumes that the overall factor structure is the same, but that item loadings and thresholds may differ across groups/time. The metric model restricts factor loadings so that they are equivalent across groups and time, and the scalar model restricts the loadings and thresholds to make them likewise equivalent. In accordance with recommendations for ordinal data, thresholds were constrained to be equal across groups/time in a separate step following the configural model. As recommended by Chen [29], a change of greater than 0.010 in Δ CFI, supplemented by a change in Δ RMSEA smaller than 0.015, was regarded as indicative of non-invariance. Missingness was 13% for IES-27 and BSL-23 at baseline and 24.9% and 22.7% at the end of treatment. Missingness was associated with younger age at both time points. Simulation studies of MI testing indicate that the WLSMV estimator (without auxiliary variables) produces relatively unbiased parameters and standard error estimates even with 50% MAR missingness when the sample size is 1000 or more [30]. All analyses were run using R version 4.2.2 (R Foundation, www.r-project.com) with the package “semTools” [31]. This study´s analysis plan was preregistered; see 10.17605/OSF.IO/KWT4Q.

## Results

### Factor structure of IES-27 and BSL-23

For the IES-27, a three-factor solution (mapping on *emotional dysregulation*, *relationship distress*, and *suicidal/ self-injury behavior*) best represented clinical symptoms in the EFA, and was empirically supported by parallel analysis, scree plot, and relative change in the RMSEA (Supplemental Material). Three items (6, 18, and 27) were removed due to absence of factor loading, and one item (9) was removed due to significant cross-loadings. Hence, only 23 of the original 27 items remained in the EFA solution. In a CFA with the 23-item version, the CFI = 0.979 and TLI = 0.977 indicated a good model fit, and the RMSEA = 0.081 [0.078; 0.084] indicated a reasonable model fit for the three-factor structure (χ^2^ = 2526.5, *df* = 227). As in previous studies, we also tested the three-factor model against a bifactor model with one general factor and the same three other factors [18]. The RMSEA decreased by -0.011 and the CFI and TLI decreased by − 0.019 and − 0.028, respectively. For further analyses, we opted for the three-factor model because of the deterioration in the CFI and TLI, the slight improvement in the RMSEA, and its greater parsimony. The following analyses are based on the three-factor model with 23 items. Composite reliability in the form of McDonald’s coefficient omega was ω = 0.88 (*suicidal and self-injury behavior*), ω = 0.88 (*relationship distress*), and ω = 0.92 (*emotional dysregulation*).

For the BSL-23, the CFI = 0.970 and the TLI = 0.967 in the CFA indicated a good model fit, and the RMSEA = 0.094 [0.092; 0.096] indicated a reasonable model fit for the one-factor model (χ^2^ = 6471.7, *df* = 230). Factor loadings ranged from 0.461 to 0.850. Composite reliability in the form of McDonald’s coefficient omega was ω = 0.94.

### Measurement invariance

For sex, the RMSEA for the configural MI was just above the 0.080 cutoff for both the IES-27 and the BSL-23, while the CFI and TLI indicated a good model fit (Table 2). Consequently, we assume that configural measurement invariance is present. After equating thresholds, factor loadings, and intercepts, we did not observe substantial change in fit indices for the two instruments.

For the IES-27 and BSL-23 (Table 3), we found a good fit for all models across time for both men and women. Moreover, model fit did not substantially decrease when constraints were added, and we could confirm strict longitudinal factor MI.

**Table 2:** Sex measurement invariance cross-sectional

Model	χ^2^	df	RMSEA	CFI	TLI	ΔRMSEA	ΔCFI	ΔTLI
IES-27								
Configural	5070.1	454	0.082	0.979	0.976			
Threshold	5139.1	500	0.078	0.979	0.979	− 0.004	0.000	0.003
Metric	5130.3	520	0.076	0.979	0.980	− 0.002	0.000	0.001
Scalar	5361.6	540	0.077	0.978	0.979	0.001	− 0.001	− 0.001
*BSL-23*								
Configural	6626.1	460	0.094	0.971	0.968			
Threshold	6651.5	506	0.089	0.971	0.971	− 0.005	0.000	0.003
Metric	6753.5	528	0.088	0.971	0.972	− 0.001	− 0.000	0.001
Scalar	7067.7	550	0.088	0.969	0.972	0.001	− 0.002	0.000

*IES-27* Impulsivity and Emotion Dysregulation Scale, *BSL-23* Borderline Symptom List, *CFI* comparative fit index, *RMSEA* root-mean-square error of approximation, *df*  degrees of freedom, ΔCFI ≥ 0.010 and ΔRMSEA ≥ -0.0015 indicate substantial deterioration in model fit. Models are compared with the prior model consisting of one less level of constraint

**Table 3:** Measurement invariance across time

				Men							Women			
Model	χ^2^(df)	RMSEA	CFI	TLI	ΔRMSEA	ΔCFI	ΔTLI	χ^2^(df)	RMSEA	CFI	TLI	ΔRMSEA	ΔCFI	ΔTLI
				IES-27							IES-27			
Configural	2997.4 (951)	.064	.980	.979				10047.3 (951)	.059	.979	.977			
Threshold	3025.8 (997)	.063	.981	.980	.001	.001	.001	10128.9 (997)	.058	.979	.978	-.001	.000	.001
Metric	3059.7 (1017)	.062	.980	.980	.000	.000	.000	10255.2 (1017)	.058	.979	.979	.000	.000	.001
Scalar	3138.9 (1037)	.062	.980	.980	.000	.000	.000	10848.8 (1037)	.059	.978	.978	-.001	-.001	-.001
				BSL-23							BSL-23			
Configural	3167.9 (965)	.066	.984	.983				13589.7 (965)	.069	.979	.977			
Threshold	3202.0 (1011)	.064	.984	.984	-.002	.000	.001	13648.0 (1011)	.068	.979	.978	-.001	.000	-.001
Metric	3231.4 (1033)	.064	.984	.984	.000	.000	.000	13729.6 (1033)	.067	.978	.978	-.001	-.001	.000
Scalar	3417.8 (1055)	.066	.983	.983	.002	.001	-.001	14919.3 (1055)	.069	.976	.977	.002	-.002	-.001

*IES-27* Impulsivity and Emotion Dysregulation Scale, *BSL-23* Borderline Symptom List, *CFI* Comparative fit index, *RMSEA* root-mean-square error of approximation, *df* degrees of freedom, ΔCFI ≥ 0.010 and ΔRMSEA ≥ -0.0015 indicate substantial deterioration in model fit. Models are compared with the prior model consisting of one less level of constraint

## Discussion

We examined MI on two self-report measures of BPD regarding sex and time in a clinical inpatient sample. Both the BSL-23 and the IES-27 were able to demonstrate MI between men and women and over time. The MI between men and women in both self-report measures is consistent with the result obtained from self-report measures collected in community samples [17]. In the BSL-23, the manifest variables loaded on a single factor representing BPD symptoms. The IES-27 differentiates between three factors according to DSM criteria: emotional dysregulation, relationship distress, and suicidality/self-harm.

In structured interviews, a lack of MI was found for the BPD criteria of impulsivity (factor loading and thresholds) and affective instability (thresholds) between men and women in an epidemiological sample [12]. For both self-report instruments, we did not find indices of variation in thresholds or factor loading for the items that assessed affective instability on the IES-27 (e.g., item: *My feelings changed rapidly between bad temper*,* anger*,* fear*,* loneliness*,* and sadness* load in the factor *emotional dysregulation*) and the BSL-23 (item: *My mood rapidly cycled in terms of anxiety*,* anger*,* and depression* load in the BPD factor). Sharp et al. [13] investigated sex-related DIF in the BPD criteria using structured interviews in an inpatient sample and found that men were more likely than women to be assigned the impulsivity and uncontrolled anger items at the same level of latent trait, while Hoertel et al. [32] found that men were less likely to report affective instability, suicidality/self-mutilation behavior, and chronic feelings of emptiness in an epidemiological sample. In the MI framework with increasingly restricted CFA models, a consistent result would be a difference in thresholds between men and women. We could not replicate these findings in patients’ self-reports. Items in the IES-27 measuring uncontrolled anger (e.g., item: *I was so angry that I could hardly control myself*) or suicidality and self-injury behavior (e.g., item: *I hurt myself by superficially cutting or scratching myself*) were endorsed at the same threshold by men and women.

Overall, the results indicated that men and women treated in an inpatient setting understand and express BPD-related problems similarly, which may suggest that potential “gender bias” is more likely to arise when external raters assess BPD-related problems. It can be observed that, in structured interviews, the BPD criteria that show sex differences in MI are those related to emotion regulation (impulsivity, affective instability; [12, 13, 32]).

If the thresholds for BPD criteria differ between men and women, it suggests that the probability of selecting a specific response category, as judged by external raters, varies by sex. We did not find this effect for thresholds in self-report instruments. One explanation may be that patients can perceive and report their internal processes more accurately, while external raters are affected by stereotypical ideas about the expression and regulation of emotions in men and women, which may lead to a lower or higher threshold for categorizing these as BPD symptomatology [33].

Furthermore, we found MI for the BSL-23 and IES-27 over time in both men and women, which is consistent with results obtained from the interview version of the ZAN-BPD in a sex-mixed clinical sample [19]. The finding of MI may be explained by the fact that most of the patients had probably already been diagnosed with BPD, and psychoeducation about the diagnosis had already taken place in previous treatments, so that the patients already had an understanding of the underlying construct prior to therapy. The same applies to recalibration.

### Limitations

Several limitations should be considered. First of all, the present sample only included men who were already in the mental-health services system. Testing MI in men from other treatment settings, such as the criminal justice system (where a higher prevalence of BPD in men is suspected) is still needed [7]. The same applies to MI between women and men from other treatment settings. In addition, patients with recent suicide attempts and current substance abuse were not included in the present sample. Men with BPD are more likely than women to be diagnosed with antisocial and narcissistic personality disorder [34, 35]. In our sample, the proportion of men with one of these additional diagnoses was 0.5% and 8.8%, respectively. Secondly, no distinction was made between gender identity and biological sex upon admission to the clinic. Accordingly, we are unable to retrospectively determine how many cases there were in which gender identity differed from biological sex, or capture patients who identified as non-binary. Furthermore, no information was collected in the routine data on whether the gender identity or biological sex (through a sex-assignment surgery) of the participants had changed over their lifetime, nor was any information gathered as to their sexual orientation. The last point is important because case-vignette studies have shown that therapists were more likely to diagnose BPD in men who were perceived as homosexual or bisexual than in those seen as heterosexual. In contrast, no effect of sexual orientation was found for women [10]. Thirdly, we possess only the patient’s self-reported data rather than an external assessment (i.e., interviews or expert’s ratings), which precludes verification of the concordance of single BPD symptoms. The use of independent raters would be preferable, as people with gender dysphoria or those who identify as the opposite sex or non-binary could experience gender minority stress, which mimics BPD and can lead to erroneous conclusions [36]. Fourthly, we did not find any evidence for measurement variance for either the BSL-23 or the IES-27 over time; however, these instruments should also be tested in a clinical sample where BPD is being diagnosed and treated for the first time.

## Conclusion

Findings suggest that the BSL-23 and the IES-27 measure BPD symptomatology similarly in men and women, and equally well before and after treatment. Thus, both instruments may be used by clinicians for the assessment of borderline symptoms in both men and women, and for comparisons before and after treatment. For researchers, both self-report instruments make it possible calculate parameters (i.e., means) and compare results; for example, in treatment efficacy studies. Future studies should examine MI in the BSL-23 and IES-27, where a greater proportion of men suffer from BPD and other personality disorders.

## Supplementary Information

Supplementary Material 1.

## Data Availability

Our data involving clinical participants are not freely available in the manuscript, supplementary files or in a public repository.
