# Supplementary material for: Measurement invariance on two self-report instruments for men and women with borderline personality disorder
Source: Borderline Personal Disord Emot Dysregul. 2025 May 12;12:17. doi: 10.1186/s40479-025-00296-1 (PMC12067672; doi:10.1186/s40479-025-00296-1)
Supplement: Supplementary file 1 — Supplementary Material 1. [file 40479_2025_296_MOESM1_ESM.docx]

**Supplementary Materials**

*Additional file 1*


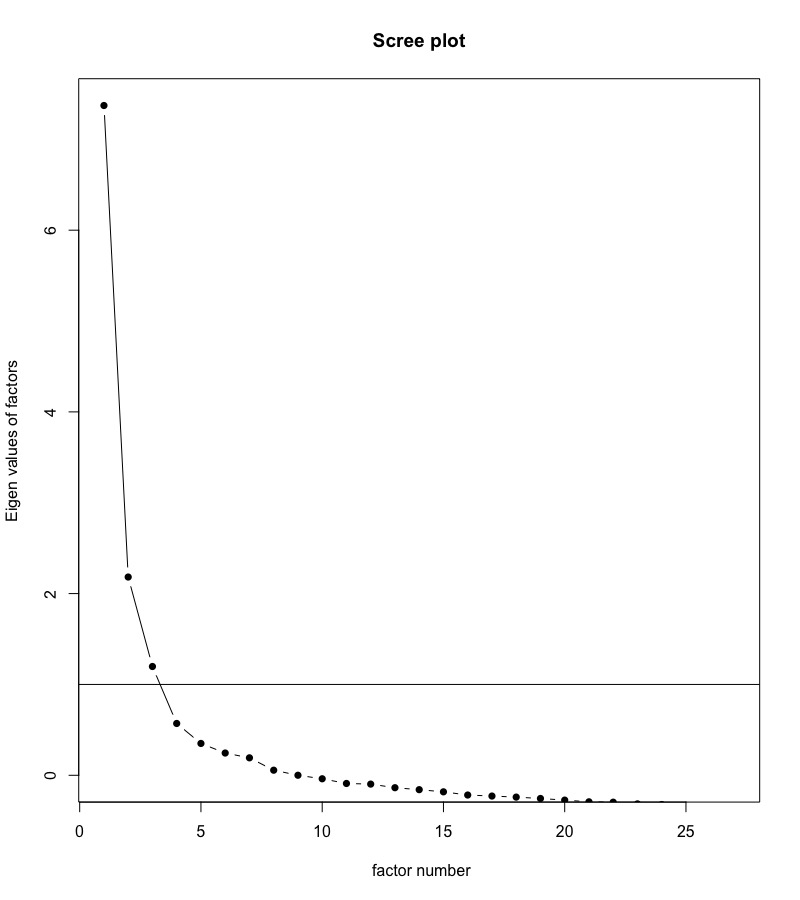


Figure S1. Screen plot of the Impulsivity and Emotion Dysregulation Scale (IES-27). The screen plot suggests a 3-factor solution.


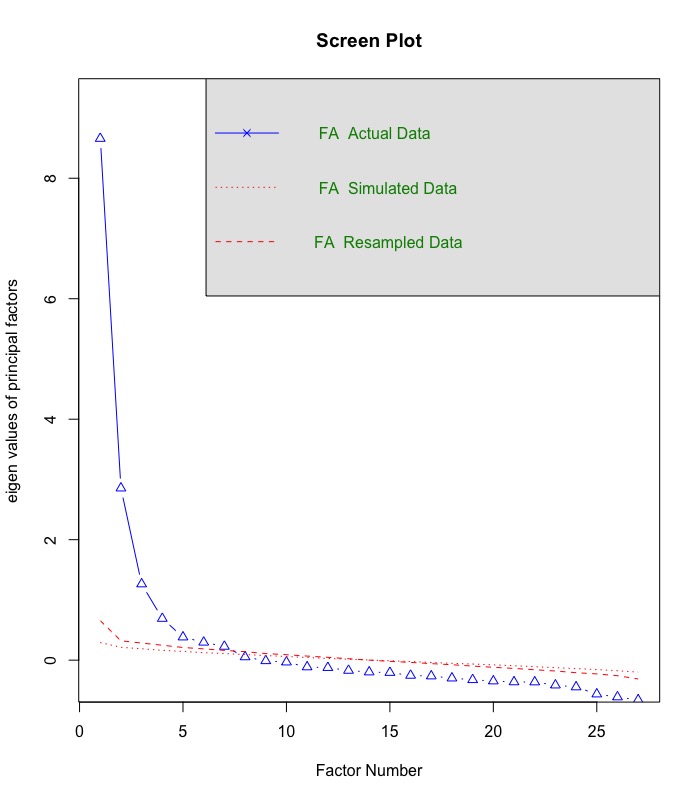


Figure S2. Parallel analysis of the Impulsivity and Emotion Dysregulation Scale (IES-27). The parallel analysis suggests the first three factors are likely to be the most important in explaining the total variance. The eigenvalue of the fourth and fifths factor were above, but very close to that generated from simulated random data.

|  | T1 | T2 | T3 |
| --- | --- | --- | --- |
| Highest Eigenvalue | 8.008 | 3.061 | 1.821 |
| Cumulative Explained variance | .302 | .450 | .408 |
| EFA factor loadings | $\lambda_{k1}$ | $\lambda_{k2}$ | $\lambda_{k3}$ |
| 1. I was afraid of losing control over my feelings | . | .62 | . |
| 1. I hurt myself by consciously knocking my head, my arm or other parts of my body against something | . | . | .343 |
| 1. I hurt myself by superficially cutting or scratching myself | . | . | .395 |
| 1. I was afraid of losing control over my actions. | . | .411 | . |
| 1. I was so angry that I could hardly control myself. | .676 | . | . |
| 1. I had eating binges. | . | . | . |
| 1. I prepared to attempt suicide. | . | . | .509 |
| 1. My feelings went up and down like a roller coaster. | . | .850 | . |
| 1. I acted very erratic | .410 | .353 | . |
| 1. I had an argument with someone. | .734 | . | . |
| 1. I had fantasies of revenge. | .408 | . | . |
| 1. I was thinking of means and ways to kill myself. | . | . | .806 |
| 1. My feelings changed rapidly between bad temper, anger, fear, loneliness and sadness. | . | .636 | . |
| 1. My relationships were constantly up and down. | .562 | . | . |
| 1. I did something without considering the consequences for me or for someone else. | .529 | . | . |
| 1. I thought about killing myself | . | . | .806 |
| 1. I experienced intense hatred | .426 | . | . |
| 1. I hurt myself by burning myself | . | . | . |
| 1. I was overwhelmed by my feelings | . | .796 | . |
| 1. I was very tense and under high pressure | . | .636 | . |
| 1. I hurt another person’s feelings, which I regretted afterwards | .724 | . | . |
| 1. I had trouble with other people | .695 | . | . |
| 1. I consoled myself with fantasies of suicide | . | . | .807 |
| 1. My emotions were muddled. | . | .883 | . |
| 1. I hurt myself by cutting myself deeply. | . | . | .431 |
| 1. I was angry | .538 | . | . |
| 1. I vomited | . | . | . |

*Additional file 2*

Exploratory Factor Analysis of the IES-27

*Note*: All factor loadings were significant *p* <0.01.

*Additional file 3*

Fit indices across the exploratory factor analysis of the IES-27

| Model | chi-square | df | rmsea | rmsea.ci lower | rmsea.ci upper |
| --- | --- | --- | --- | --- | --- |
| 1-factor | 4017.046 | 324 | 0.087 | 0.085 | 0.089 |
| 2-factor | 1782.428 | 298 | 0.057 | 0.055 | 0.060 |
| 3-factor | 742.232 | 273 | 0.034 | 0.031 | 0.037 |
| 4-factor | 461.966 | 249 | 0.024 | 0.020 | 0.027 |

*Note.* IES-27 = Impulsivity and Emotion Dysregulation Scale, RMSEA = root-mean-square error of approximation, *df* = degrees of freedom

*Additional file 4*

Factor loadings BSL-23

| Model | CFA Model  λ | Configural  Model  λ | |
| --- | --- | --- | --- |
| BSL-23 items |  | Men | Women |
| 1. It was hard for me to concentrate | .525 | .630 | .506 |
| 1. I felt helpless | .713 | .712 | .714 |
| 1. I was absent-minded and unable to remember what I was actually doing | .550 | .554 | .552 |
| 1. I felt disgust | .574 | .573 | .575 |
| 1. I thought of hurting myself | .669 | .622 | .678 |
| 1. I didn´t trust other people | .555 | .529 | .564 |
| 1. I didn´t believe in my right to live | .738 | .756 | .738 |
| 1. I was lonely | .630 | .659 | .626 |
| 1. I experienced stressful inner tension | .647 | .709 | .633 |
| 1. I had images that I was very much afraid to | .578 | .687 | .558 |
| 1. I hated myself | .850 | .836 | .852 |
| 1. I wanted to punish myself | .791 | .788 | .792 |
| 1. I suffered from shame | .655 | .588 | .668 |
| 1. My mood rapidly cycled in terms of anxiety, anger and depression | .693 | .745 | .684 |
| 1. I suffered from voices and noised from inside or outside my head | .461 | .549 | .451 |
| 1. Criticism had a devastating effect on me | .587 | .593 | .584 |
| 1. I felt vulnerable | .636 | .678 | .625 |
| 1. The idea of death had a certain fascination for me | .600 | .559 | .616 |
| 1. Everything seemed senseless to me | .742 | .718 | .748 |
| 1. I was afraid of losing control | .649 | .626 | .654 |
| 1. I felt disgusted by myself | .769 | .705 | .782 |
| 1. I felt as if I was far away from myself | .601 | .623 | .598 |
| 1. I felt worthless | .825 | .834 | .824 |

*Additional file 5*

Factor loadings IES-27

| Model | CFA Model  λ | Configural  Model  λ | |
| --- | --- | --- | --- |
| IES-27 items |  | Men | Women |
| Suicidal and self-injury behaviour |  |  |  |
| 2. I hurt myself by consciously knocking my head, my arm or other parts of my body against something | .523 | .538 | .547 |
| 3. I hurt myself by superficially cutting or scratching myself | .482 | .530 | .519 |
| 7. I prepared to attempt suicide. | .778 | .731 | .730 |
| 12. I was thinking of means and ways to kill myself. | .882 | .901 | .899 |
| 16. I thought about killing myself | .922 | .910 | .919 |
| 23. I consoled myself with fantasies of suicide | .873 | .901 | .879 |
| 25. I hurt myself by cutting myself deeply. | .562 | .501 | .616 |
| Emotional dysregulation |  |  |  |
| 1. I was afraid of losing control over my feelings | .803 | .803 | .801 |
| 4. I was afraid of losing control over my actions. | .746 | .717 | .750 |
| 8. My feelings went up and down like a roller coaster. | .870 | .873 | .867 |
| 13. My feelings changed rapidly between bad temper, anger, fear, loneliness and sadness. | .803 | .836 | .800 |
| 19. I was overwhelmed by my feelings | .861 | .878 | .842 |
| 20. I was very tense and under high pressure | .708 | .740 | .697 |
| 24. My emotions were muddled. | .862 | .889 | .856 |
| Relationship distress |  |  |  |
| 5. I was so angry that I could hardly control myself. | .799 | .804 | .809 |
| 10. I had an argument with someone. | .638 | .587 | .685 |
| 11. I had fantasies of revenge. | .519 | .559 | .496 |
| 14. My relationships were constantly up and down. | .733 | .672 | .732 |
| 15. I did something without considering the consequences for me or for someone else. | .636 | .528 | .633 |
| 17. I experienced intense hatred | .687 | .708 | .674 |
| 21. I hurt another person’s feelings, which I regretted afterwards | .668 | .640 | .685 |
| 22. I had trouble with other people | .645 | .623 | .668 |
| 26. I was angry | .748 | .729 | .751 |
